# The Role of MicroRNAs in Development of Endometrial Cancer: A Literature Review

**DOI:** 10.18502/jri.v24i3.13271

**Published:** 2023

**Authors:** Somasundaram Indumati, Birajdar Apurva, Gaur Gaurav, Singh Nehakumari, Vyas Nishant

**Affiliations:** 1- Department of Stem Cell and Regenerative Medicine, D.Y. Patil Education Society, Kolhapur, India; 2- Logical Life science, Maharashtra, India

**Keywords:** Biomarkers, Endometrial neoplasms, Gene therapy, MicroRNAs, Signal transduction

## Abstract

Endometrial cancer (EC) ranks as the second most common gynaecological cancer worldwide. EC patients are diagnosed at an early clinical stage and generally have a good prognosis. Therefore, there is a dire need for development of a specific marker for early detection of endometrial adenocarcinoma. The development of EC is conditioned by a multistep process of oncogenic upregulation and tumor suppressor downregulation as shown by molecular genetic evidence. In this setting, microRNAs appear as significant regulators of gene expression and several variations in the expression of microRNAs have been implicated in normal endometrium, endometrial tissue, metrorrhagia, and endometrial cancer. Furthermore, microRNAs act as highly precise, sensitive, and robust molecules, making them potential markers for diagnosing specific cancers and their progression. With the rising incidence of EC, its management remains a vexing challenge and diagnostic methods for the disease are limited to invasive, expensive, and inaccurate tools. Therefore, the prospect of exploiting the utility of microRNAs as potential candidates for diagnosis and therapeutic use in EC seems promising.

## Introduction

Endometrial cancer (EC), a common female genital cancer, is also known as uterine corpus cancer or corpus cancer (1). World wide, it is the second most common gynaecological cancer, with an estimating 417,367 new cases and 97,370 deaths annually (2). Essentially, EC is one of only two common cancers that does not follow the general inclination of progress in morbidity and death with a worse survival rate today than in the 1970s when it hit a peak in 1975 in parts of the US (3, 4). Risk factors include aging, hormonal imbalance, and obesity and these are some of the factors that cause a rise in the incidence of endometrial cancer. Yet, genetic factors significantly influence EC ranging from the inheritance of various low-risk cancer susceptibility variants to single and very high-risk variants (5). Based on histologic and pathologic characteristics, EC is divided into two subtypes, namely endometrioid (Type I) and non-endometrioid (Type II). Patients with Type I are usually diagnosed with early-stage disease and generally have a good prognosis. However, patients with Type II succumb to poorly differentiated or high-grade cancer with the worst prognosis (6, 7). Endometrioid adenocarcinoma (EAC) has emerged as the most common subtype, amounting to approximately 75% of EC cases (8). In patients diagnosed with advanced-stage or recurrent EAC, the survival rate is low due to the inefficacy of treatments (9). These subtypes exhibit distinctive mutations, amplification, and overexpression profiles though they share some similarities (10). Conversely, there are four molecular subtypes of endometrial carcinoma, as identified through The Cancer Genome Atlas (TCGA): DNA polymerase ε exonuclease domain mutation, microsatellite-instable, microsatellite-stable with copy-number low, and microsatellite-stable with copy-number high (10). Four prognostic groups are defined as follows:

1) Polymerase-E (POLE) ultra-mutated: Patients exhibit the most promising prognosis and the longer progression-free survival. Genes that show mutations associated with POLE ultra-mutated are POLE, PTEN, PIK3R1, PIK3CA, FBXW7, KRAS, and TP53 (11). In EC patients, the most common alterations identified in POLE were P286R and S297F in exon nine and V411L, L424V, and L424I in exon thirteen (12).
2) Microsatellite instability hypermutated: Patients exhibit moderate prognosis. Associated genes with alterations are PTEN, KRAS, and ARID1A (11).
3) Low copy-number: These patients also show moderate prognosis. Associated genes are CTNNB1 and PTEN (11).
4) High copy-number: Unfortunately, these patients suffer the worst prognosis. The most common histotype is serous. Mutated genes observed in this group are TP53, FBXW7, and PPP2R1A (11).

The development of EC is conditioned by a multistep process of oncogenic upregulation and downregulation of tumor suppressor as shown by molecular genetic evidence (13). The central genes associated with endometrial oncogenesis or progressions are K-ras, B-raf, Her2/neu, β-catenin, AKT, and FGFR2 oncogenes (14). The genes involved in the loss-of-function events are phosphatase and tensin homolog (PTEN), p53, and genome instability-derived genes (14). The most common genetic alteration is in the PTEN gene, which acts as a tumor suppressor gene and such alterations are the most frequent genetic lesions in Type II. In general, 25%–83% of tumors were reported in PTEN mutations, more often in endometrioid carcinomas and microsatellite unstable tumors (15). In addition to PTEN, genetic variations in p53, another tumor suppressor gene, were observed in 25% of all ECs (11). A type of genomic instability, telomere shortening, was observed in both Type I and Type II ECs (14). Furthermore, Type I and II exhibited differential DNA methylation patterns, thus proposing different epigenetic backgrounds and unique carcinogenic pathways (14).

EC develops in the inner lining of the uterus called the endometrium. It is a highly dynamic tissue that undergoes recurrent proliferative and secretory changes with every menstrual cycle which makes it susceptible to genetic and epigenetic changes (16). This dynamic process is controlled by sex hormones that result in marked changes in gene expression (17). In this setting, microRNAs appear as significant regulators of gene expression and several variations in the expression of microRNAs have been implicated in normal endometrium, endometrial tissue, metrorrhagia, and EC (18). MicroRNAs are a class of small, endogenous RNAs with a length of 21–25 nucleotides that function as major players of post-transcriptional gene regulation in diverse species. It has been observed that expression profile of microRNAs displays considerable dysregulation during carcinogenesis (19, 20). Many studies have shown the role of microRNAs as post-transcriptional regulators involved in cell proliferation, invasion, angiogenesis, apoptosis, and resistance to treatment (21–23). Additionally, through post-transcriptional regulation of important chromatin- and DNA-modifying enzymes, microRNAs have also been associated with the control of chromatin structure and gene expression (24). Growing evidence has also shown that microRNAs are associated with tumorigenesis and progression as oncogenes or tumor suppressor genes (25). This information highlights the multifaceted roles of microRNAs regarding their association with cancer and its types. Further, microRNAs act as highly precise, sensitive, and robust molecules, making them potential markers for diagnosing specific cancers and their progression (26–29). The fact that diagnostic and screening tools of EC can be invasive, expensive, and/or inaccurate, and that the aetiology and prognostic factors are not fully known, the prospects of exploiting the utility of microRNAs as potential candidates for therapeutic use in EC seem promising. Therefore, the objective of this review was to explore the potential diagnostic value of microRNAs in EC with the intend to elucidate and provide new insights into the diagnosis and prognosis of EC. It is hoped that the findings would provide novel strategies for risk assessment and help in fine-tuning therapeutic treatments targeted at EC patients and immensely improve the overall outcome for such patients.

### Synthesis and function of microRNAs:

MicroRNAs are a class of small, endogenous RNAs with a length of 21–25 nucleotides that function as major players of posttranscriptional gene regulation in diverse species (30, 31). The biogenesis of microRNAs from primary microRNAs is completed in two stages, by the action of two RNase III proteins, Drosha in the nucleus and Dicer in the cytoplasm (32). Immature microRNAs undergo further modifications in order to become mature microRNAs; they are processed by Dicer and RNase III protein and added to Argonaute (Ago) protein so as to form the effector RNA-induced silencing complex (RISC). RISC contains the mature microRNA duplex, which further synchronizes the translation of complementary mRNA and escorts it to target microRNA (33, 34). The correspondent sequences in the 3′ untranslated region (3′-UTR) are identified by mature microRNA via seed region (35, 36).

A microRNA does not completely bind to the complementary target mRNAs. Thus, one micro-RNA can regulate a variety of genes (usually around 500), influencing expression of many target genes (37). Therefore, microRNAs bear various roles, comprising the regulation of protein-coding genes expression and translation suppression at transcriptional level and post-transcriptional level, respectively (38). Additionally, various physiological and pathological studies on protein production, gene regulation, chromatin variation, and tumorigenesis have been conducted with a focus on oncogenic or tumor suppressor factors (39–40).

### MicroRNAs as gene regulators in endometrial cancer:

**Figure 1.**
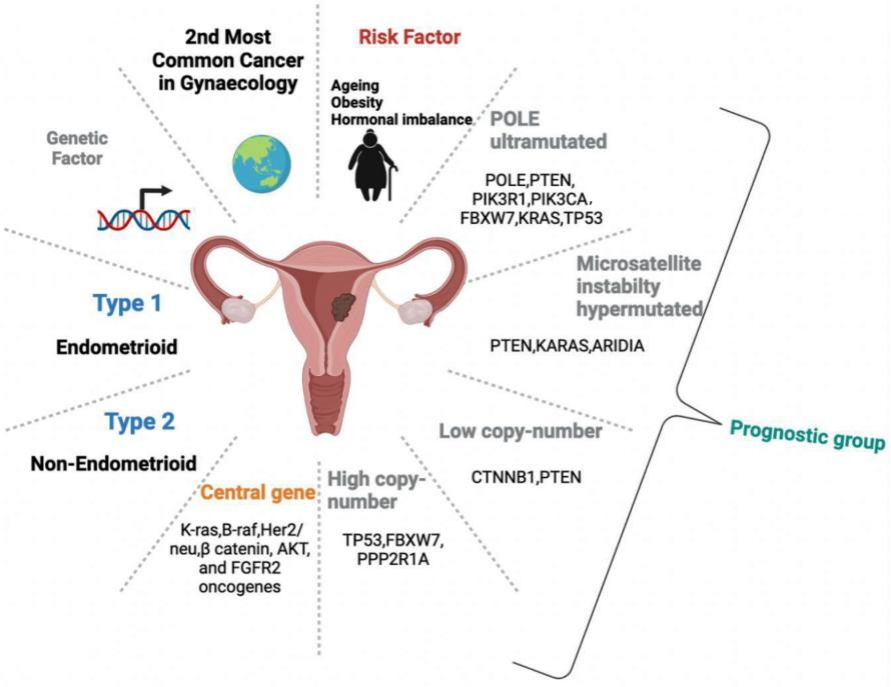
The figure shows EC is a sophisticated system, depicting its occurrence, categorization, prognostic groups, and oncogenic genes

The discovery of microRNAs, especially mutations affecting microRNA function, has led to the extensive understanding of the pathogenesis of various types of diseases including cancer (41). Calin et al. in 2002 described the first association of microRNAs and cancer in chronic lymphocytic leukaemia, where patients exhibited low expression of two microRNAs, miR-15 and miR-16 (42). As mentioned, the role of microRNA in cancer is either to function as oncogenes or tumor suppressors. Overexpression of microRNAs can inhibit tumor suppressors or related genes involved in cell differentiation, thus leading to the formation of tumors through proliferation, angio-genesis, and invasion (oncogenes). Correspondingly, microRNAs can act as tumor suppressors by hindering oncogenic activity of different proteins (43). MicroRNAs are involved in many tumor-related steps like tumor cell invasion and metastasis including epithelial–mesenchymal transition (EMT) and systemic circulation (44). EMT is a regulatory mechanism which significantly controls metastasis in EC (45). Several studies have delved into the roles of microRNAs in EMT and cell metastasis. For instance, it was shown that overexpression of miR-23a could inhibit EMT by targeting SMAD3 in endometrial cancer (46). It has been reported that EC is a multifactorial disease with numerous anomalous steps in gene expression process (47). Several studies have shown the association of 313 micro-RNAs where 48 microRNAs were downregulated, 140 were upregulated, and 22 had inconsistent expression. It was also observed that some microRNAs were highly common in EC, namely miR-182, miR-183, miR-200, and miR-205 (48–51) (Figure 1).

### MicroRNAs as epigenetic modifiers in endometrial cancer:

**Figure 2.**
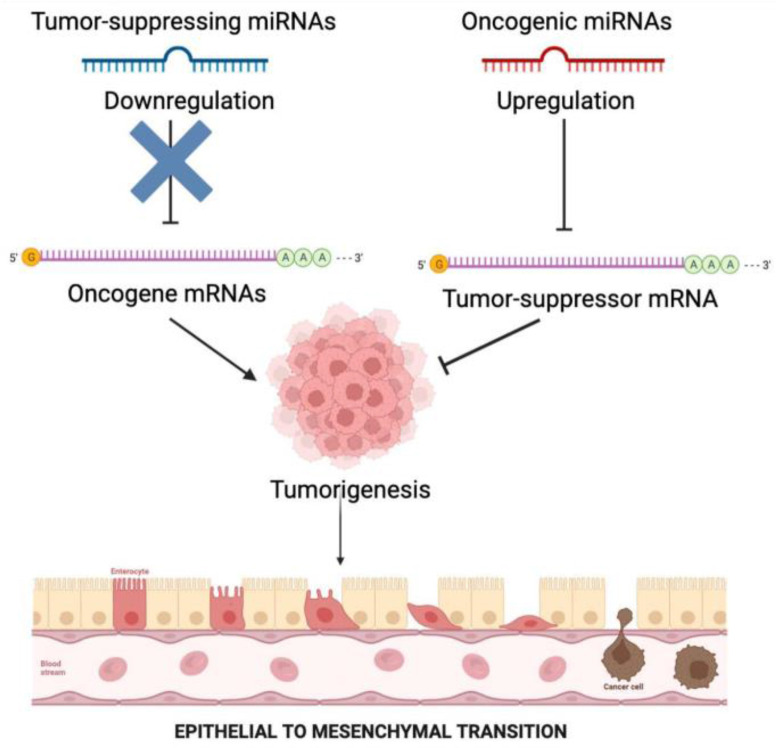
MicroRNAs act as oncogenes or tumor suppressors in cancer. Overexpression of microRNAs may block tumor suppressors or associated genes involved in cell differentiation, leading to tumor growth, angiogenesis, and invasion (oncogenes). MicroRNAs are involved in tumor invasion, metastasis, EMT, and systemic circulation

Favier et al. in 2021 provided an outline of microRNAs involved in epigenetic regulation in EC including DNA methylation and RNA silencing. The conclusions described in their study may lead to innovative approaches in diagnosis, determining risk factors, and treatments targeted at microRNAs, their target genes, or DNA methylation (52) (Figure 2):

1) MicroRNAs have been identified to act as tumor suppressors or as oncogenes. For example, the inhibition of apoptosis occurs as a consequence of direct binding of miR-200 to PTEN. Inhibition of cell growth is initiated by miR-182 direct binding to LRIG2 while increase in cell proliferation occurs due to miR-182-mediated decline in cullin-5 protein levels.
2) CpG-rich domains of miRNA loci can be hypoor hyper-methylated. For instance, downregulation of miR-129-2 expression occurs due to hypermethylation, leads to activation of SOX4 oncogene, and this microRNA inhibits SOX4 under normal conditions.
3) MiR-191 inhibits Tet Methylcytosine Dioxygenase 1 (TET1), a protein coding gene, through the mRNA-microRNAs interaction in the 3′-untranslated region of TET1. This induces hypermethylation of the promotor region of adenomatous polyposis coli (APC), a tumor suppressor, ensuing reduced gene expression and lessened levels of APC protein.

### Key microRNAs as main players in endometrial cancer:

Many studies have shown an aberrant expression of microRNAs in EC and such aberration has been associated with tumorigenesis (53–54). A few microRNAs as key players involved in regulatory events in EC are described hereunder; these microRNAs may be useful in characterizing the nature of EC, help in understanding the related risk factors, and tailor therapeutic processes of EC (Table 1).

**Table 1.** Differential expression of microRNAs in endometrial cancer, their target genes, and signaling pathways

**MicroRNAs**	**MiRNA upregulated**	**MiRNA downregulated**	**Target gene**	**Signalling pathway**
**MiR-202**	NO	YES	β-catenin, fibroblast growth factor 2 (FGF2)	Wnt/β-catenin pathway
**MiR-501**	YES	NO	HOXD10, a member of the Abd-B homeobox family	AKT/mTOR pathway
**MiR-302a-5p/367-3p**	NO	YES	High mobility group AT-hook 2 (HMGA2)	Wnt/β-catenin pathway
**MiR-29b**	NO	YES	Phosphatase and tensin homolog (PTEN)	MAPK/ERK and PI3K/AKT signalling pathways
**MiR-199**	NO	YES	FAM83B gene (a family with sequence similarity 83, member B)	PAK4/MEK/ERK signalling pathway
**MiR-135**	YES	NO	Phosphatase and tensin homolog (PTEN) and protein kinase B (p-AKT)	MAPK/ERK and PI3K/AKT signalling pathways
**MiR-137**	NO	YES	Elevated polycomb group protein enhancer of zeste homolog 2 (EZH2) and lysine-specific histone demethylase 1 (LSD1)	Notch 1 signalling pathway
**MiRNA-200**	NO	YES	Zinc finger E-box binding homeobox 1 and 2 (ZEB1 and ZEB2); PTEN	MAPK/ERK and PI3K/AKT signalling pathways
**MiRNA-205**	NO	YES	Zinc finger E-box binding homeobox 1 and 2 (ZEB1 and ZEB2); PTEN	MAPK/ERK and PI3K/AKT signalling pathways
**MiR-181**	YES	NO	Transforming growth factor (TGF-β), TGF-β receptors, estrogen receptor alpha (ERα), ERβ and PR	TGF-β signalling pathway

**Figure 3.**
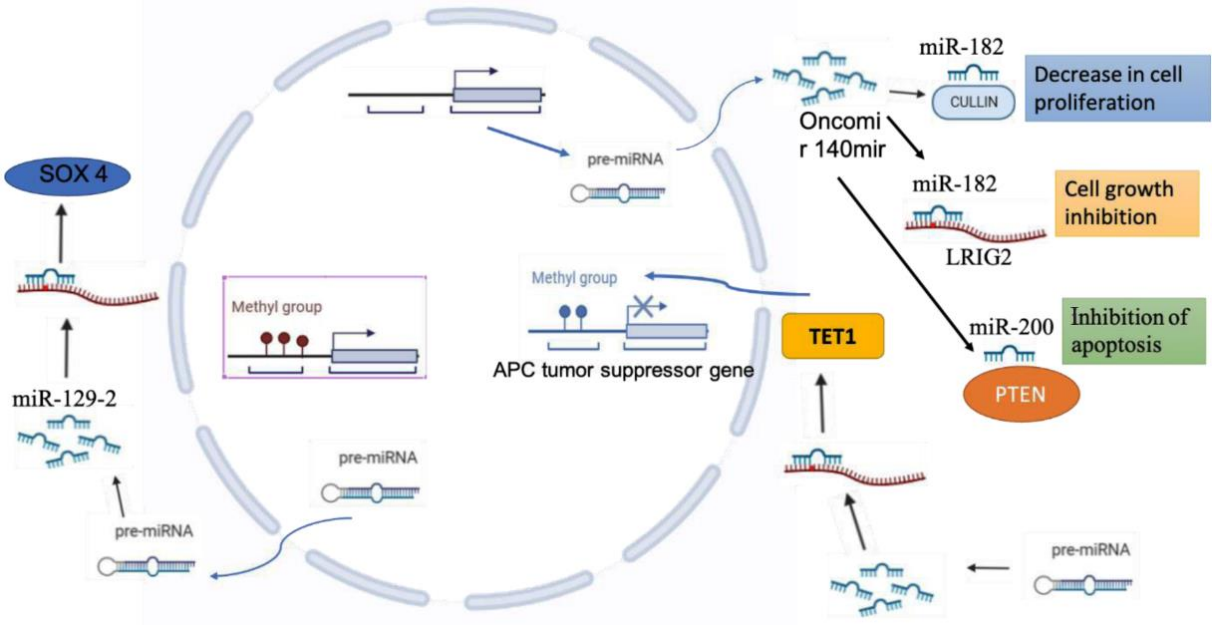
A diagram of the epigenetic processes that are known to be involved in microRNAs formation: DNA methylation and RNA-associated silencing are outlined for the microRNAs implicated in epigenetic regulation in EC

**Figure 4.**
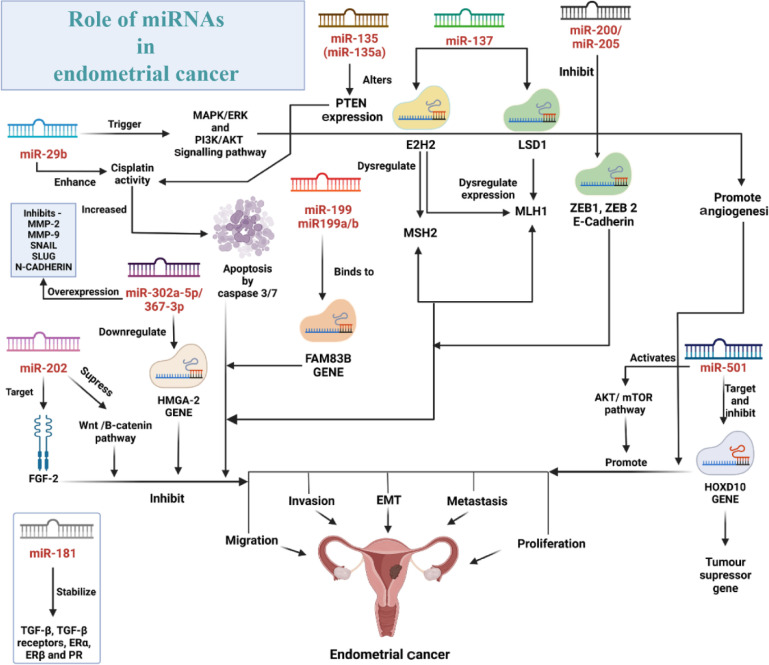
The figure illustrates a concise image of a complex microRNA network and its role in the progression and amelioration of EC with a detailed representation of signalling pathways, proteins, and genes involved

1) MiR-202: Chen et al. in 2019 showed the downregulation of miR-202 in EC tissues, which was related with aggressive behaviors in EC patients. MiR-202 had an inhibitory effect on migration, invasion, and EMT in EC (55). The study showed that the overexpression of miR-202 inhibited the Wnt/β-catenin pathway by suppressing β-catenin expression in EC. The dysregulation of the pathway plays a significant role in development, progression, and cell metastasis of EC (56, 57). Additionally, it was also seen that fibroblast growth factor 2 (FGF2) was directly targeted by miR-202 and thus, suppressed migration and invasion efficiently through targeting FGF2 in EC. Subsequently, considering the regulatory role of miR-202 in EC cell metastasis, the aberration of miR-202 expression can consequently be used as a potential marker for EC (Figure 3).

2) MiR-501: Sun et al. in 2021 observed that copy number high EC exhibited an overexpression of miR-501 when compared to the benign endometrial tissues, and such overexpression indicates poor survival in EC patients (58). When over-expressed, miR-501 leads to advancement of migration, tissue invasion, metastasis (pelvic lymph node metastasis), and shorter overall survival. The mechanism of such regulatory roles of miR-501 involves targeting a tumor suppressor gene, HOXD10, a member of the Abd-B homeobox family, and encoding a protein with a homeobox DNA-binding domain. Therefore, overexpression of miR-501 inhibits HOXD10 expression; however, miR-501 activates the AKT/mTOR pathway. The aberrant expression of HOXD10 has been observed in various cancers, such as ovarian cancer, prostate cancer, endometrial cancer, esophageal squamous cell carcinoma, colon cancer, and glioblastoma through regulatory activities like metastasis in cancers and tumor cell proliferation (59–64). The differentiation of a group of high-grade tumors with low survival rate and evaluation of the risk of pelvic lymph node metastasis is facilitated by the detection of miR-501 in EC samples; therefore, the miR-501 might be a potential therapeutic target (62) (Figure 4).

3) MiR-302a-5p/367-3p: Ma et al. in 2018 showed the altered expression of miR-302a-5p/367-3p and its association with EC malignant cells through the downregulation of high mobility group AT-hook 2 (HMGA2) expression (65). HMGA2 acts as an oncogene and promotes tumor growth and metastasis (66). The study showed that the knockdown of HMGA2 suppressed tumorigenesis in EC cell lines, Ishikawa and HEC-1A cells, while upregulation of HMGA2 induced malignancy of these cells, specifying an oncogenic role of HMGA2 in EC. The expression of EMT-related proteins, such as MMP-2, MMP-9, Snail, Slug, and N-cadherin was suppressed, while the expression of E-cadherin was amplified. The miR-302a-5p/367-3p was overexpressed and HMGA2 was reduced in EC cells. Thus, HMGA2 is a functional target of miR-302a-5p and miR-367-3p. Therefore, this regulatory association of miR-302a-5p/367-3p and HMGA2 can be considered as a predictive therapeutic target in EC (65) (Figure 4).

4) MiR-29b-3p: Kong et al. in 2019 stated that in endometrial cancer, miR-29b-3p expression is suppressed and it is associated with poor prognosis (67, 68). Upregulation of miR-29b-3p lessened the proliferation of HEC-1-B and Ishikawa EC cells and enhanced cisplatin sensitivity. Cisplatin is an effective anticancer drug used in EC treatment (69, 70). Overexpression of miR-29b-3p increased cisplatin-induced apoptosis by enhancing activities of caspase 3/7. The study also showed that the upregulation of miR-29b-3p reduced migration and invasion of HEC-1-B cells and Ishikawa cells. MiR-29b-3p triggered AKT pathway by targeting PTEN in EC cells. Chen et al. found that miR-29b-3p was involved in angio-genesis in EC by targeting MAPK/ERK and PI3K/AKT signalling pathways (71). As a result, miR-29b-3p could act as a prognostic marker for the clinical response to chemotherapy (67) (Figure 4).

5) MiR-199a/b-5p: This microRNA is a highly conserved microRNA family that includes miR-199a and miR-199b (72). Xiong et al. in 2021 showed that the expression of miR-199a/ b-5p was suppressed in human EC cells compared with human endometrial epithelial cells (HEECs). Through the inhibition of EMT- associated factors and EMT signalling pathway, miR-199a/b-5p repressed the migration and invasion of the EC cells (72). Conversely, FAM83B gene, a novel bio-marker of various cancers including gastric cancer (73) and pancreatic ductal adenocarcinoma (74), lessened such miR-199a/b-5p regulatory activities through upregulation of EMT signalling pathway. MiR-199a/b-5p repressed EMT by attaching to 672–679 nucleotides of the 3′-UTR of FAM83B. The study showed the association of FAM83B and miR-199a/b-5p in the regulatory network of EMT, which increases the development of EC metastasis. Consequently, the simultaneous investigation of miR-199a/b-5p and FAM83B may improve the accuracy and precision of strategies in cancer diagnosis (72) (Figure 4).

6) MiR-135a: Wang et al. in 2020 observed that the proliferation of both HEC-1-B and Ishikawa EC cells was either increased or decreased by up or downregulation of miR-135a via regulation of EMT-related proteins expression (75). Additionally, the expression profiles of PTEN and p-AKT in EC cells were altered by miR-135a. It has been studied that up to 55% of sequence anomalies in PTEN have been identified in EC (76). Cisplatin is an effective drug in EC treatment. The study implicated an association between the expression level of miR-135a with cisplatin induced apoptosis in EC cells by targeting AKT signaling pathway. Hence, such association may be indicative of the effect of miR-135a on increasing the sensitivity of EC cells to treatment with cisplatin. In summary, miR-135a’s role in tumor-igenesis and EC progression by modulating proliferation and fine tuning chemotherapy of EC cells is indicative of its potential as a biomarker for diagnosis and prognosis in EC (75) (Figure 5).

7) MiR-137: Zhang et al. showed that in both endometrioid endometrial carcinoma (EEC) and serous endometrial primary tumors, miR-137 was highly hypermethylated compared to normal endometrium (77). This suggests a regulatory role of miR-137 (downregulation) in endometrial tumorigenesis. This epigenetic methylation of miR-137 was related with microsatellite instability (MSI) which is initiated by alteration of DNA mismatch repair genes such as MLH1, MSH2, PMS1, and PMS2 (77). Their analysis suggested that miR-137 inhibited EC by supressing cell proliferation and targeting elevated polycomb group protein enhancer of zeste homolog 2 (EZH2) and lysine-specific histone demethylase 1 (LSD1) proteins, therefore, acting as a tumor suppressor agent. EZH2 and LSD1 played regulatory roles in MLH1 expression (78, 79) where overexpression of EZH2 led to inhibition of MSH2 expression (80). These outcomes propose that methylation of miR-137 is indirectly related with MSI status. Subsequently, miR-137 has diagnostic properties and can be used as an intermediate marker of EC in diagnosis and treatment (77) (Figure 4).

8) MiR-200: Snowdon et al. in 2011 specified the overexpression of the miR-200 family expression in EEC despite the fact that most cancers have presented a suppression of the same microRNA. Studies have shown that the miR-200 family has been associated with suppression of EMT (81–84). Two transcription factors, namely, zinc finger E-box binding homeobox 1 and 2 (ZEB1 and ZEB2), are inhibited by miR-200 family, consecutively inhibiting E-cadherin. It is observed that with low miR-205 expression, these two transcription factors are upregulated and E-cadherin is decreased and cells exhibit a mesenchymal phenotype with increased capacity in tissue invasion and metastasis. Low expression of miR-200 is also related with an aggressive tumor phenotype (85, 86). Investigations proved that estrogen receptor α (ERα) and estradiol control regulatory activities of the miR-200 family (87). Cases where endometrial cancers exhibited ERα expression showed a reduced amount of aggressiveness in tumors than those devoid of ERα expression (88). This is associated with an overexpression of miR-200 family; as a consequence, it aids in the maintenance of epithelial phenotype, thereby hindering the transition of EMT (81) (Figure 4).

9) MiR-205: MiR-205 may act as a tumor suppressor or an oncogene in carcinogenic cases; miR-205 may be one of the microRNAs that is crucial in determining tumor proliferation and invasion (89). Wilczynski et al. in 2016 described that the patients were diagnosed with better prognosis when miR-205 was highly expressed. In conjugation with miR-200 family, miR-205 was reported to control expression of the E-cadherin transcriptional repressors, ZEB1 and ZEB2, as factors that were key players in EMT (89). Gregory et al. revealed that in cells which exhibited EMT, transforming growth factor (TGF–β) inhibited miR-205 expression, showing that miR-205 inhibition through overexpression of ZEB1/ZEB2 may activate EMT stimulation which is crucial in EC progression (82). MiR-205 achieves the regulatory influence by targeting proteins such as PTEN suppressor gene, demonstrating an inverse relationship with EC where inactivation is one of the mostly reported genetic aberrations in EEC (occurring in 37–61% of cases) (90, 91). In early stage and advanced EC cases where miR-205 is upregulated, PTEN is negatively regulated (92–93). It has already been proved that miR-205 negatively regulates PTEN expression post-transcriptionally (91, 92). Wilczynski et al. indicated in their studies that good prognosis, better survival rate, and fewer aggressive stages are related with an overexpression of miR-205 (95) (Figure 4).

10) MiR-181a: From several studies on the aberrant expression of miR-181a, it can be concluded that miR-181a has the potential to be used as a biomarker for risk assessment and clinical therapy in multiple cancers such as colorectal cancer (CRC), breast cancer, squamous cell carcinoma, and EC (94–95). Intrinsically, the stability of TGF-β, TGF-β receptors, ERα, ERβ, and PR and other target genes, is influenced by such aberrant expression of miR-181a in EC; as a consequence, local expression profiles vary and therefore, the outcome of endometriosis will be changed. Endometrial tumors exhibit altered profiles of endometrial expression of miR-181a and miR-98 (96). MiR-181a enhances migration and invasion in cells by regulating EMT as observed in epithelial ovarian cancer (EOC) via the TGF-β signalling pathway (97). Additionally, functional analysis of these microRNAs demonstrated their association with cellular apoptosis, differentiation, cell communication, and carcinogenesis with their genes confined to sites of repeated chromosomal instability (98) (Figure 4).

### Major signaling pathways mediated by microRNAs in EC:

Important signalling pathways are involved in the multistage development of EC mediated by many microRNAs to regulate several genes involved in relevant biological processes. Therefore, investigations should be conducted to clarity and study the potential mechanisms of EC tumor-igenesis and development. Further, the need to identify key pathogenic factors for better prognosis and improved clinical outcome is also stressed simultaneously. Clinical trials could be conducted on the drugs which attack these pathways and are effective in the treatment of EC.

1) PI3K/AKT/mTOR signaling pathway: Studies have shown that aberrations in this pathway are very frequent in endometrial carcinoma and its precursor lesions may progressively result in unfortunate medical outcomes (99–102). The low expression of PTEN, a well-known tumor suppressor, regulates the activation of this oncogenic pathway in EC (103). PTEN mutation is exhibited in the very beginning of EEC, existing in 20%–27% of endometrial hyperplasias (104, 105) and in 55% of endometrial intraepithelial neoplasias (106) and these mutations occur before mismatch repair defects in the growth of irregular EEC cells (107). Mutations in the members of PI3K pathway, such as PIK3CA, PIK3R1 (p85α), or PIK3R2 (p85β), can functionally imitate the loss-of-function in PTEN, and consequently enhance the p-AKT levels (108). In general, 43% of endometrioid ECs and 12% of non-endometrioid ECs displayed PIK3R1 mutation (109). MicroRNAs are key players in regulating a wide range of genes and signals involved in controlling EMT events (110, 111). MiR-21 andmiR-205 inhibit PTEN expression via binding to the 3′-untranslated region (3′-UTRs) of PTEN mRNA; therefore, cell growth of EC cells increases and indicates poor prognosis (112). MiR-21 regulates AKT expression through suppression of PTEN via a double-negative feedback loop. It has been observed that miR-21 inhibits PTEN which in turn reduces AKT activity, resulting in the overexpression of miR-21 (113). Several microRNAs namely miR-7 (114), miR-99a, miR-100, and miR-101 were shown to influence mTOR in tumor cells. It has been shown that EC cell lines devoid of PTEN are sensitive to PARP inhibitors, indicating a prospective marker for targeted therapy in EC (115).

2) Wnt/β-catenin signaling pathway: In EEC, Wnt/β-catenin signaling pathway is the second most commonly activated pathway (116) due to its key role in several cellular events such as cell growth, differentiation, and maintenance of pluripotency (117). Many growth-related pathologies and cancer types are associated to the aberrant Wnt pathway components (118). The key downstream effector of this pathway is β-catenin which is encoded by CTNNB1 gene and this mutation of the gene has been observed in endometrial hyperplasia (119). The translocation of the β-catenin protein from the membrane to the nucleus leads to activation of Wnt/β-catenin signaling which occurs due to an aberrant CTNNB1 gene expression (120–122). It has been demonstrated that microRNAs modulate EMT by aiming at stimulating the Wnt/β-catenin signaling pathway (123). For instance, an upregulation of miR-200 targets ZEB1 and ZEB2, which enhances E-cadherin expression and hinders EMT. The growth of EC is enhanced by miR-373 through activation of Wnt/β-catenin pathway and large tumor suppressor kinase 2 (LATS2) (124).

3) Notch signalling pathway: Numerous cancer types and associated tumorigenesis have displayed an aberrant Notch signalling (125). Amongst other pathways, the Notch pathway is one of the signalling pathways that modulates tissue development and proliferation, differentiation, and apoptosis in various cell types (126, 127). Four Notch transmembrane receptors (NOTCH 1–4) and five transmembrane ligands, including three Delta-like proteins (DLL1, DLL2, and DLL4) and two jagged proteins (JAG1, JAG2), are well known in mammals (9). An activated Notch intracellular domain (NICD) is released from the plasma membrane via ligand binding and when the NICD reaches the nucleus, it functions as a transcriptional activator and increases the expression of related genes (128). An alteration in the activation of the Notch signalling pathway is associated with increased cell growth in various cancers, including endometrial adenocarcinoma (129, 130). Jurcevic et al. showed that the aberrant expression of miR-34a causes the alteration in the expression of NOTCH1, a notch protein coding gene of the Notch family and delta like canonical notch ligand 1 (DLL1), a protein coding gene in EAC (9). However, Gao et al. described that miR-134 inhibited EC stem cells by targeting protein oglucosyltransferase 1 (POGLUT1) and Notch proteins (131).

4) RAS-RAF-MEK-ERK signalling pathway: An aberrant RAS-RAF-MEK-ERK signalling pathway is commonly exhibited in human cancers. KRAS has emerged as the most common mutation in human cancers amongst the three RAS isoforms (132), and KRAS activation occurs early at the very beginning of endometrial carcinogenesis (133). In EC, KRAS mutations concur with PTEN, PIK3CA, and PIK3R1 mutations, suggesting that KRAS mutations share a functional synergy with PI3K pathway mutations (134, 135). Studies have shown that miR-143 negatively regulates proliferation, migration, and invasion of EC cells by inhibiting MAPK1 (136). MiR-29b causes negative regulation of angiogenesis and tumor growth in EC by downregulating vascular endothelial growth factor A (VEGFA) via deactivation of the MAPK/ERK and PI3K/AKT pathways (137).

5) Janus Kinase/signal transducers and activators of transcription (JAK-STAT) signaling: JAKSTAT pathway has been observed to play an important role in tumor progression (138). In EC cells, the alteration of JAK-STAT pathway results in enhanced cell proliferation (139). MiR-20a-5p, miR-124, and miR-361 have a negative influence on cellular processes like migration, invasion, and EMT by targeting STAT3 gene (140–142).

### MicroRNAs as diagnostic biomarkers and therapeutic agents:

The incidence of EC is rising and diagnosis pivots on invasive tests with no screening tool for either general or high-risk population groups. Although exhaustive research has been conducted on cancers such as ovarian and cervical, the same must be warranted in EC (143). At present, the use of biomarkers is necessary for diagnosis of EC. In recent times, many studies have explored the prospective of utilizing micro-RNAs as diagnostic and therapeutic tools (144).

Exploration of tissue specific patterns of micro-RNAs expression could source potential markers for tracing the root of a cancer, which is fundamental in providing an effective treatment plan. As the diagnosis of cancer is associated with the expression profiles of numerous microRNAs, these profiles of microRNAs expression may act as prognostic indicators (18). Boren et al. observed in their study that 13 microRNAs (hsa-let-7i, hsa-miR-221, hsa-miR-30c, hsa-miR-152, hsamiR-193, hsa-miR-185, hsa-miR-106a, hsa-miR-181a, hsa-miR-210, hsa-miR-423, hsa-miR-103, hsa-miR-107, and hsa-let-7c) and 90 mRNAs resulting from human EC and normal uterine tissues were involved in EC proliferation, and suggested that the 13 microRNAs recognized 26 (29%) of the 90 mRNAs as targets. A total of 11 of these 26 mRNA genes (KCNMB1, IGFBP-6, ENPP2, TBL1X, CNN1, MYH11, KLF2, TGFB1/1, MYL9, SNCAIP, and RAMP1) were shown to be related to and responsible for cell growth (145). With several studies in EC, up to 754 microRNAs (146) have been recognized as prospective biomarkers in EC, some of which show a link with lymph node metastasis, advanced FIGO stage, and tissue invasion (147–150). Another example of microRNA as a non-invasive marker is the combination of both miR-199a and miR-542-3p which can be used to detect endometriosis, a precancerous lesion of EC that is an indicator for early diagnosis with a sensitivity of 96.61% and specificity of 79.66% (151).

Another advantage of microRNAs is that they are highly stable in extracellular fluids of mammals, such as serum, blood plasma, and urine. Urine appears to be a favorable non-invasive test for the detection of EC; nonetheless, only in one study, urine was employed for the detection of microRNAs (152, 153). Tan et al. found that in a study of miR-155 in serum, elevation of miR-155 was associated with EC stage and lymph node metastases (154). Torres et al. showed a high probability for EAC diagnosis by using a combination of four serum microRNAs, miR-222, miR-223, miR-186, and miR-204, in serum as per a genome-wide study (155).

Subsequently, microRNAs expression profiles vary in cancer cells; in fact, suppression of the upregulation of an oncogene type microRNA or promotion of the downregulation of a tumor suppressor-type microRNA may have a therapeutic effect (18). For instance, in EC, some microRNAs such as miR-423, miR-103, miR-205, miR-429, and miR-135a are upregulated and act as oncogenes influencing tumorigenesis, cell growth, and progression (18, 156) while some down-regulated microRNAs like miR-221, miR-193, miR-30c, and miR-99b in EC act as tumor suppressors (18). It has been observed that microRNAs are differentially expressed in EC as compared with normal endometrium; the same is true when endometrioid and serous papillary EC is compared, indicating the mechanisms that are specific to individual tumor subtypes (157). In endometrioid EC, neoplastic processes like migration, invasion, and survival can be calibrated by targeting microRNA expression via up or downregulation of the specified microRNAs. For example, the overexpression of miR-7 in EC can be inhibited by using anti-microRNAs oligonucleotides, which result in reduced migration and invasion (158). On the other hand, the level of miR-194 can be upregulated via transfection with pre-microRNAs molecule which otherwise is low in EC patients with more advanced stage and an overall poor prognosis; therefore, EMT phenotype and EC cell invasion are repressed by targeting the oncogene BMI-1 (159, 160). A number of clinical trials have been explored to evaluate the inhibition of microRNAs, comprising anti-micro-RNA oligonucleotides, microRNA sponges, microRNA masks (target protectors), and small molecule inhibitors (161). For example, the chemoresistant properties of miR-100 in NSCLC could be used in decreasing the drug resistance of tumors (162) and the epigenetic silencing of miR-199b-5p has been shown in drug resistant ovarian carcinoma (163).

Another approach for treating patients with advanced stages diagnosed with poor outcomes is to focus on EC cells by fine-tuning tumor related signaling pathways. Consequently, regulation of dysregulated microRNAs could be instrumental in tuning aberrant signaling pathways in EC. Type II EC exhibits KRAS mutation and studies have shown that the expression profiles of miR-181b, miR-324–3p, and miR-518b are decreased in cancer with a KRAS mutation (164). PTEN is a target gene for microRNA 200a, 200b (165), and 205a (166), which are involved in the PI3K/AKT/mTOR signaling pathway and are associated with poor prognosis. Studies have focused on this signaling pathway as a remedial target for therapy since cells with mutations responded positively to the use of a PI3K/mTOR inhibitor and the use of temsirolimus as an mTOR inhibitor (167). Micro-RNAs have been shown to regulate drug mechanisms and resistance. For example, bortezomib is a drug for EC treatment and its efficacy is enhanced by miR-17-5p on p21 pathway (168). A lowered expression of miR-34c regulates metastasis, apoptosis, and invasion in EC and the combination of miR-34c mimic with cisplatin enhanced the drug efficiency in cell lines (169).

The treatment of malignant tumors by using microRNAs may alter the expression of approximately 100 genes (18). Due to their stability, microRNAs can exist in various tissues and structures, and easily transport in target areas via blood vessels and other structures (170). A significant number of cancer cells and lowered expression of target genes were observed when intravenous injection of nanoparticles modified with a humanized monoclonal antibody GC4 and miR-34a and two siRNAs was administered (171). When compared to molecular targeted drugs, the effects of microRNAs are comparatively mild and cause fewer side effects in normal cells (18). This approach of treatment triumphs over the limitation of conventional gene therapy, in which only one gene is targeted, even though a malignant tumor is a composition of collective anomalies (18).

Different from gene mutations, epigenetic changes that are associated with global gene regulation such as chromatin remodeling open a new field of cancer research (172). Epigenetic silencing of tumor suppressor genes or epigenetic activation of oncogenes play important roles in the promotion of carcinogenesis and tumor progression (172).

Altered expression profiles of microRNA have been observed in EC compared with normal endometrium (173). Several microRNAs are differentially expressed in endometrioid and serous papillary EC, indicating that they could infer mechanisms that are specific to individual tumor subtypes (174). Among those microRNAs elevated in endometrioid EC, the expression of miR-7 can be downregulated by using anti-microRNA oligonucleotides, repressing migration and invasion of EC cells (175). On the other hand, the level of miR-194 was significantly lower in EC patients with more advanced stage, and lower expression of this microRNA was associated with worse survival. Moreover, overexpression of miR-194 by transfection with pre-microRNA molecule inhibited EMT phenotype and EC cell invasion by targeting the oncogene BMI-1 (160).

MicroRNAs are stable in various tissues and body fluids (176). This property greatly facilitates the delivery of microRNAs to recipient cells via the blood or other compartments. All in all, targeting those microRNAs that are deeply involved in EC progression would provide a promising therapeutic option for EC. Forced expression of tumor suppressor microRNA and suppression of oncogenic microRNA are two strategies to achieve the goal of microRNA-based cancer treatment. Although previous results demonstrated that restoration of tumor suppressor miR-152 effectively inhibited EC cell growth *in vitro* and *in vivo* (177), obvious challenges of finding efficient delivery systems and tumor cell specificity must be resolved to allow clinical implementation.

### Challenges in using microRNAs as biomarkers:

One of the main reasons restricting the use of microRNAs as biomarkers and diagnostic tools in clinical settings is that frequently reported microRNAs are found in patients with a variety of tumor types. For instance, circulating miR-21 has been reported to be upregulated in individuals with breast, colorectal, prostate, liver, esophageal, and endometrial cancer. Even in quite similar studies of the same diseases, the outcomes are inconsistent. Therefore, it seems that the relationship between the disease and microRNAs is not well established. Genetic mutation or epigenetic silencing may cause downregulation of microRNAs in tumors; however, this is only likely to occur if the tumor negatively affects the expression of the microRNAs in other cells or reduces the stability of the microRNAs in circulation. Consequently, reductions in circulating microRNAs may be explained as general responses to the presence of cancer. Furthermore, the increase of circulating microRNAs cannot be linked to tumor burden. Only a few number of cancers, such as late-stage tumors for instance, cause excessive expression of certain microRNAs due to the dilution effects of blood.

Post transcriptional modification of these diagnostic microRNAs is not well characterized which may question the stability and anonymity of processes prior to making these microRNAs. Finally, it may be more useful to use a panel of well chosen microRNAs rather than one to ensure that the biomarker is specific to cancer.

## Conclusion

Early detection of EC in people increases the chance of survival which, however, is not the case in patients in whom the cancer is detected at a rather advanced stage with a survival chance of at least 10% to 29% (15). This necessitates the urgent development of a method to detect the disease as early as possible. The discovery of new microRNAs highlights the possibility of introducing new strategies in the diagnosis, prognosis, and treatment of human cancers and the present case of EC due to their observed biological functions. These newly discovered microRNAs reveal the potential to act as biomarkers and/or therapeutic targets. Understanding the roles that they play in regulating the oncogenic processes and calibrating oncogenic signalling pathways appears to be a significant step in this regard. However, more studies have to be carried out to ascertain the clinical utility of microRNAs and select best microRNAs for the relevant clinical practices involved in this field. In fact, with the establishment of new genomic classifications of endometrial cancers, non-invasive microRNA bio-markers can play a crucial role to drive therapeutic approaches in the treatment of cancer in the future. The introduction of such approaches is hoped to reduce the adverse effects of EC and related aspects such as invasive diagnostic methods and poor prognosis. The approaches could also help in initiating early treatment for affected cases and detect the presence of EC in people, thereby significantly increasing their chances of survival.
